# Biliary Microbiota in Health and Disease: Clinical Implications in Lithiasis, Infection, and Antimicrobial Resistance

**DOI:** 10.3390/antibiotics15050445

**Published:** 2026-04-29

**Authors:** Claudia Marinaccio, Marta Giovanetti, Benedetto Neri, Dario Biasutto, Andrea D’Amico, Annamaria Altomare, Francesco Branda, Laura Restaneo, Massimo Ciccozzi, Michele Cicala, Michele Pier Luca Guarino

**Affiliations:** 1 Gastroenterology and Endoscopy Unit, Campus Bio Medico University of Rome, 00128 Rome, Italy; claudia.marinaccio@unicampus.it (C.M.); m.cicala@policlinicocampus.it (M.C.);; 2 Department of Sciences and Technology for Sustainable Development and One Health, Campus Bio Medico University of Rome, 00128 Rome, Italy; 3 Therapeutic GI Endoscopy Unit, Fondazione Policlinico Universitario Campus Bio-Medico, 00128 Rome, Italy; d.biasutto@policlinicocampus.it; 4 Unit of Medical Statistics and Molecular Epidemiology, Campus Bio Medico University of Rome, 00128 Rome, Italy; f.branda@unicampus.it (F.B.);; 5 Research Unit of Gastroenterology, Department of Medicine and Surgery, Campus Bio Medico University of Rome, 00128 Rome, Italy

**Keywords:** biliary microbiota, antimicrobial resistance, gut–liver axis, dysbiosis

## Abstract

The biliary tract, long considered a sterile environment, is now recognized to harbor a resident microbiota with important implications for health and disease. This review aims to summarize current knowledge on the composition and function of the biliary microbiota in physiological conditions, and its alterations in pathological states such as infection and lithiasis, with a particular focus on antimicrobial resistance. In healthy individuals, the biliary microbiota appears to be shaped by bile acids and gut–bile axis interactions, playing a role in local immune modulation. In disease, microbial dysbiosis contributes to conditions such as acute cholecystitis, cholangitis, and gallstone formation, with distinct microbial signatures linked to specific stone types. Common biliary pathogens, including *E. coli*, *Enterococcus* spp., *Pseudomonas* spp., and *K. pneumoniae*, often exhibit concerning resistance patterns, impacting therapeutic strategies. Emerging evidence highlights the interplay between intestinal and biliary microbiota, suggesting potential diagnostic and prognostic applications. Understanding these dynamics opens new avenues for microbiota-informed antibiotic stewardship, targeted microbiota modulation, and precision medicine approaches. Further research, particularly culture-independent and longitudinal studies, is crucial to fully elucidate the clinical significance of the biliary microbiota and to integrate microbiota profiling into patient management strategies.

## 1. Introduction

### 1.1. Anatomy of the Biliary System

The biliary tree is a complex anatomical network originating from the intrahepatic canals of Hering channeling the bile through a series of progressively larger ducts that converge into the common hepatic duct, which ultimately conveys the bile to the gallbladder and the duodenum [1]. Bile is an aqueous secretion composed of both organic and inorganic solutes. These include bile salts, cholesterol, bilirubin phospholipid, vitamins, amino acids, and enzymes [1]. It is primarily secreted by hepatocytes in the bile canaliculi with a filtration mechanism. This process is driven by the osmotic gradient generated by bile salt-dependent and independent transport systems, located on the hepatocytes apical membrane [2]. Subsequently, the bile is modified by cholangiocytes, ciliated epithelial cells lining the intrahepatic ducts, whose main function is to fluidize and alkalinize canalicular bile in response to the post-prandial release of secretin [2,3,4]. Bile flows backwards to the direction of portal blood flow, it is channeled into the canals of Hering, passing through a series of progressively larger ducts: small bile ductules (diameter < 15 μm), interlobular ducts (15–100 μm), septal ducts (100–300 μm), area ducts (300–400 μm), segmental ducts (400–800 μm), until the hepatic ducts (>800 μm) and major ducts [2,5]. Upon entering the extrahepatic ducts, the bile is carried through the common bile duct. Along its path, it joins with the cystic duct coming from the gallbladder and, further downstream, merges with the pancreatic duct. Ultimately, the bile flows to the ampulla of Vater, where it is delivered into the intestine [6]. The bile plays a crucial role in maintaining systemic homeostasis. In addition to its well-established role in facilitating the intestinal absorption of dietary fats [7], bile also contributes to the regulation of the immune system through the excretion of immunoglobulin A [8]. Moreover, it acts as a vehicle for the elimination and transport of hormones and fat-soluble vitamins, and represents the primary elimination route for both endogenous and exogenous substances [2].

### 1.2. Bile: Basic Concepts

Bile has historically been described as a sterile fluid in healthy conditions [9,10]. This concept was supported by the antimicrobial effect of bile salts, which induce DNA and membrane damage in bacteria [11]. However, in the late 1960s, Flemma et al. demonstrated the presence of bacteria in bile of asymptomatic patients without a history of cholangitis, thereby introducing the concept of “asymptomatic bactibilia” [12]. Over the past decades, the introduction of 16S ribosomal RNA sequencing and other gene sequencing techniques has facilitated further studies on bile microbiota that challenged the traditional notion of bile sterility [13,14]. However, the reliability of invasive sampling methods has always represented a relevant limitation to the investigation of bile microbiota. Bile collecting techniques such as endoscopic retrograde cholangiopancreatography (ERCP), percutaneous biliary drainage and intra-operatory sampling, are all burdened by a high risk of contamination. This methodological limitation largely accounts for the predominant focus of current literature on the role of biliary microbiota in pathological conditions, such as cholelithiasis and biliary tract infections [15,16,17]. Nonetheless, Molinero et al. described for the first time a microbial ecosystem in the bile of healthy subject by enrolling 13 liver donors without previous known hepatobiliary diseases [18]. In this study the presence of three dominant bacterial phyla in the healthy subject group (*Actinobacteria*, *Bacteroidetes*, and *Firmicutes*) alongside *Proteobacteria* was reported [18]. In light of methodological biases identified in this study, including the limited sample size and the pre-sampling administration of antibiotics, further studies are warranted to conclusively establish the composition of biliary microbiota in healthy individuals [18]. A comprehensive characterization of the biliary microbiota may help elucidating the etiopathogenesis of biliary tract disorders. This may even influence the choice of antibiotic therapy, which may require us to consider the antimicrobial resistance profiles of the resident bacterial populations [19].

The present review aims to summarize current knowledge regarding the composition and function of biliary microbiota under healthy conditions. It further investigates the potential interplay between biliary dysbiosis and the pathogenesis of lithiasis, and the major infectious diseases of the biliary tract. Considering the relevance of the theme, a particular emphasis has been placed on the role of antimicrobial resistance.

## 2. The Biliary Microbiota in Healthy Individuals

### 2.1. Composition and Diversity of the Biliary Microbiota in Non-Pathological Conditions

The dominant bacterial phyla identified in the bile of living donors or patients undergoing cholecystectomy for non-infectious conditions include *Firmicutes*, *Proteobacteria*, *Bacteroidetes*, and *Actinobacteria* [18,20,21]. At the genus level, commonly detected taxa are *Streptococcus*, *Escherichia-Shigella*, *Prevotella*, *Veillonella*, *Lactobacillus*, and *Enterococcus*. These are characterized by their capacity to tolerate bile and survive under fluctuating oxygen conditions. A detailed overview of the main microbial groups and their adaptive features is presented in Table 1. These genera are known for their bile tolerance and capacity to survive under conditions of fluctuating pH, high osmolarity, and antimicrobial bile acids. Interestingly, differences in biliary microbial composition compared to the gut microbiota suggest selective pressures unique to the biliary environment [21]. Although bacteria dominate, preliminary evidence indicates that fungi (notably *Candida* species) and bacteriophages may also form part of the biliary ecosystem, although their precise roles remain unclear [22]. In healthy conditions, the biliary microbiota appears relatively stable, but technical challenges, such as low microbial biomass and potential contamination during sample collection, require careful interpretation of findings. Notably, inter-individual variability exists, potentially influenced by host factors such as age, diet, genetics, bile acid composition, and anatomical integrity of the biliary system (e.g., the functionality of the sphincter of Oddi) [22].

### 2.2. Role of Bile Acids in Modulating Microbiota

Bile acids are key modulators of the biliary microbiota. Synthesized from cholesterol in hepatocytes, primary bile acids (such as cholic acid and chenodeoxycholic acid) are conjugated with taurine or glycine to increase their solubility before being secreted into bile. In the duodenum, bile acids facilitate lipid digestion, but they also exert potent antimicrobial activity. The amphipathic structure of bile acids allows them to integrate into bacterial membranes, causing membrane destabilization, leakage of cellular contents, and inhibition of bacterial growth [23]. However, certain microbial species have evolved mechanisms to resist bile toxicity. For example, many commensal bacteria express bile salt hydrolase (BSH) enzymes that deconjugate bile acids, reducing their detergent properties and enabling bacterial survival [24]. Other adaptations include modifications to membrane composition and the activation of efflux pumps. Bile acids also function as signaling molecules. They interact with host receptors such as the farnesoid X receptor (FXR) and G protein-coupled bile acid receptor 1 (GPBAR1 or TGR5), regulating metabolic pathways, immune responses, and barrier functions [25]. In turn, microbial metabolites can modify bile acid pools, creating a feedback loop between microbiota and host bile acid metabolism. Importantly, secondary bile acids, such as deoxycholic acid and lithocholic acid, are products of gut microbial metabolism and can return to the biliary tract via enterohepatic circulation. These modified bile acids further influence biliary microbiota composition, favoring bile-resistant species and possibly contributing to long-term homeostasis or dysbiosis depending on the host and environmental context [23].

### 2.3. Gut–Bile Axis: Translocation, Enterohepatic Circulation, and Immune Interactions

**Figure 1:**
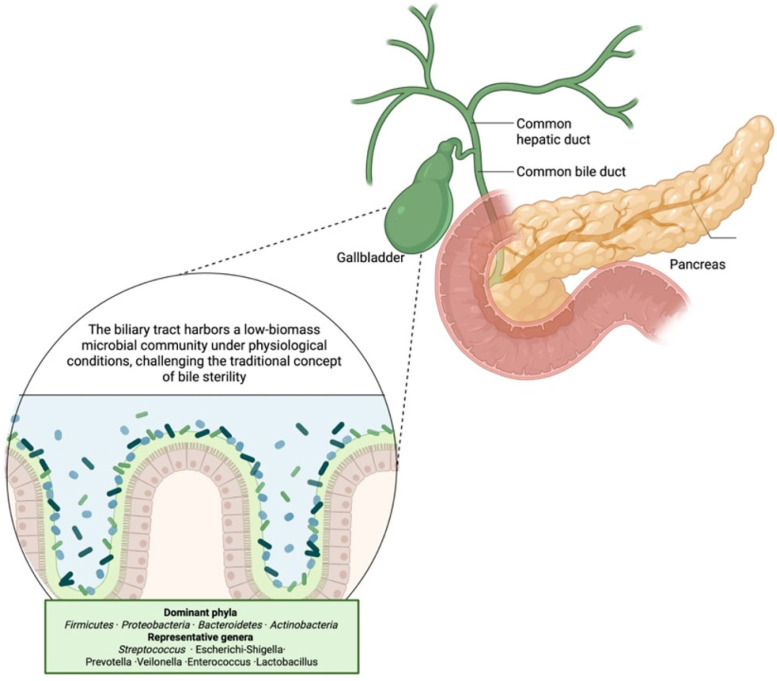
Composition of the biliary microbiota and its relationship with the intestinal microbial ecosystem. Contrary to the traditional view of biliary sterility, emerging sequencing-based studies reveal the presence of a low-biomass but diverse microbial community within bile. Dominant bacterial phyla include *Firmicutes*, *Proteobacteria*, *Bacteroidetes*, and *Actinobacteria*. Representative genera frequently identified in bile samples include *Streptococcus*, *Escherichia-Shigella*, *Prevotella*, *Veillonella*, *Enterococcus*, and *Lactobacillus*. These taxa display adaptive mechanisms enabling survival in bile-rich environments and reflect the close ecological relationship between intestinal and biliary microbial niches. Created in BioRender. Restaneo, L. (https://www.figurelabs.ai/, accessed on 3 March 2026) is licensed under CC BY 4.0.

The concept of the gut–bile axis highlights the close anatomical and functional relationship between the gastrointestinal tract, liver, and biliary system. This axis facilitates microbial and molecular exchanges, contributing to physiological regulation but also creating potential routes for pathogenic insult when barriers are disrupted [26]. Translocation of microbes or microbial products from the gut into the portal circulation is a recognized phenomenon. Low-level, controlled microbial migration may contribute to immune education and tolerance in the liver and the biliary tree. The sphincter of Oddi, functioning as a mechanical barrier, normally prevents duodenal content reflux into the bile duct. However, intermittent micro-leakage may allow selective microbial seeding without triggering inflammation under healthy conditions [26]. Enterohepatic circulation ensures that approximately 95% of bile acids secreted into the duodenum are reabsorbed in the ileum and returned to the liver. This recycling exposes the biliary system to gut-derived microbial metabolites, including secondary bile acids and short-chain fatty acids (SCFAs), which can influence cholangiocyte biology and immune signaling. Immune interactions in the biliary tract are finely tuned to maintain tolerance toward commensal microbiota while providing defense against pathogens. Cholangiocytes express a range of pattern recognition receptors (e.g., TLR2, TLR4, NOD-like receptors) that recognize microbial-associated molecular patterns (MAMPs) [25]. Activation of these receptors triggers downstream signaling pathways leading to the production of antimicrobial peptides, cytokines, and chemokines. Moreover, secretory IgA transported through biliary epithelial cells contributes to neutralizing microbes without inducing inflammation, promoting balanced microbiota. Resident immune cells, such as Kupffer cells in the liver and peribiliary lymphocytes, further contribute to the surveillance and maintenance of biliary homeostasis [25]. Dysregulation of these mechanisms may lead to excessive immune activation, inflammation, and biliary tract disease. The interactions between bile microbiota and intestinal microbial system are summarized in Figure 1.

## 3. Alterations of the Biliary Microbiota in Disease

### 3.1. Infectious Conditions of the Biliary Tract

Acute cholecystitis and cholangitis represent the most common infectious diseases of the biliary tract. These conditions are typically subsequent to biliary obstruction caused by gallstones, leading to overgrowth of pathogenic bacteria [27]. In acute cholecystitis the obstruction of the cystic duct by gallstones generates an acute inflammatory response leading to bacterial infection in 20% of patients [28]. In 1990 Thompson et al. identified preoperative temperature > 37.3 °C, total serum bilirubin level higher than 8.6 μmol/L, and white blood cell count greater than 14.1 × 10^9^/L as predictors of bactibilia [29]. Similarly, cholangitis involves impaired bile flow which results in elevated intraductal pressure facilitating ascending infection and hematogenous translocation of bacteria from the gastrointestinal tract through the portal venous system [30]. On the other hand, biliary obstruction can induce bacterial translocation from bile to the bloodstream, potentially leading to a systemic inflammatory response and sepsis, which are associated with mortality rates ranging between 10–20% [31,32]. Acute biliary infections may cause a rapid deterioration of clinical conditions, particularly in older patients with comorbidities [32,33]. Hence, it is crucial to initiate antibiotic therapy as soon as a definitive diagnosis is established, or promptly in patients presenting with shock [33]. The current literature shows a higher prevalence of infections caused by Gram-negative bacteria, particularly members of the Enterobacteriaceae family [30,34,35,36,37]. Gram positive cocci, in particular *Enterococcus* spp. may also be present in hemocultures and bile cultures [38]. The most prevalent Gram-negative isolates are *E. coli*, *K. pneumoniae*, and *P. aeruginosa*. Among Gram-positive pathogens, the species most frequently identified are *E. faecium*, *E. avium*, and *E. faecalis*. With regard to fungal isolates *C. albicans*, *C. tropicalis* and *C. parapsilosis* seems to be the more frequently isolated [39,40]. In some cases, anaerobic bacteria, such as *B. fragilis* and *C. perfringens* can also account for the development of cholangitis [30].

Empirical antibiotic therapy should be based on the clinical context, including symptoms’ severity, patient’s age and allergies, infection’s onset (community or hospital-acquired) and local antibiotic resistance [41]. According to the latest guidelines, it is recommended to perform blood cultures and bile cultures, when patients undergo biliary drainage, possibly before starting empirical antibiotic therapy, to avoid the induction of multi-resistant strains selection [33].

**Figure 2:**
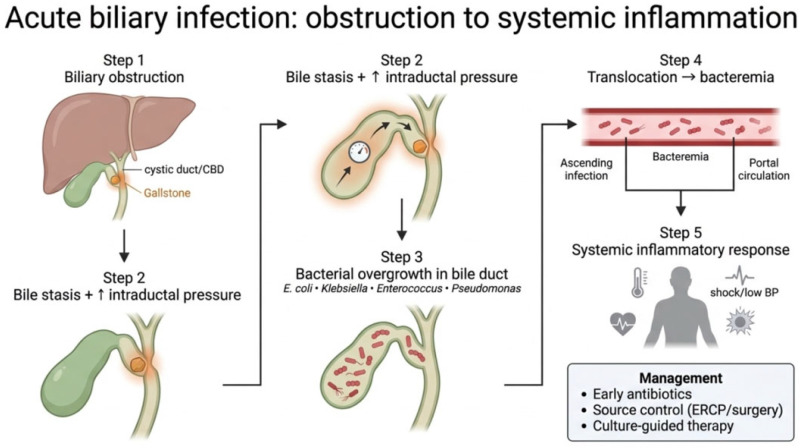
Pathophysiological cascade linking biliary obstruction to systemic infection. Obstruction of the biliary tract, most frequently caused by gallstones, induces bile stasis and increased intraductal pressure, creating a favorable niche for bacterial proliferation within the bile ducts. Enteric pathogens—including *E. coli*, *Klebsiella* spp., *Enterococcus* spp., and *Pseudomonas* spp.—can expand locally and subsequently translocate into the bloodstream, leading to bacteremia and systemic inflammatory response. If untreated, this process may progress to sepsis and hemodynamic instability. Early recognition and prompt management, combining antibiotic therapy with source control through biliary drainage (e.g., ERCP or surgery), are critical to interrupt this pathogenic cascade. Created in BioRender. Restaneo, L. (https://www.figurelabs.ai, accessed on 3 March 2026) is licensed under CC BY 4.0.

Overall, the most common antimicrobial agents chosen are represented by beta-lactam antibiotics, covering entero-bacteria, frequently associated with metronidazole to cover anaerobic bacteria. *Enterococci* should be covered in case of nosocomial infection, high severity, or immunodepression. Fluoroquinolones are usually not recommended due to antibiotic resistance, particularly in western countries, and to their poor efficacy against *Enterococci* and *Streptococci* [30]. An antibiotic therapy duration of 4 to 7 days after control of the source of infection is recommended, with the exception for *enterococci* and *streptococci* which require longer treatments due to the risk of endocarditis [42]. The correct management of a patient with acute biliary infection, including surgical and/or endoscopic interventions, may help prevent complications such as acute pancreatitis (particularly in case of lithiasic etiology), septic shock, abscesses and gallbladder perforation [30]. Figure 2 summarizes the cascade linking biliary obstruction to systemic infection.

### 3.2. Biliary Lithiasis and Gallstones

The isolation of bacteria from the bile of patients with gallstone has prompted to explore the microbiological component of gallstone formation. While infections may arise secondary to biliary tract obstruction, bacteria can also access the biliary system via the sphincter of Oddi or through hematogenous spread via the portal circulation [43,44]. Microbiological analysis of gallstones revealed a higher abundance of the genera *Actinomyces*, *Streptococcus*, and *Achromobacter* compared to bile, suggesting a potential role of these microorganisms in gallstone formation [45]. In recent years, a significant role of microorganisms in the pathogenesis of gallstones has been reported, particularly through biofilm formation and production of specific bacterial enzymes [46,47]. β-glucuronidase and phospholipase have been indeed isolated from bile cultures. The first prevalently promotes the hydrolysis of bilirubin diglucuronides, leading to the formation of unconjugated bilirubin, which can subsequently precipitate as calcium bilirubinate [48]. This enzymatic deconjugation may facilitate the formation of calcium bilirubinate crystals, especially when associated with anionic glycoproteins, ultimately contributing to their aggregation into pigment gallstones [49]. Differently, phospholipase degrades lecithin in the bile, increasing the concentration of lysolecithin and free fatty acids. These compounds promote the formation of a lithogenic core, which serves as a nidus for bile salt crystallization and aggregation. Bacterial phospholipase activity on biliary phosphatidylcholine can lead to the release of palmitic acid, which subsequently binds to ionized calcium to form calcium palmitate. The precipitation of calcium palmitate results in solid complexes that may become incorporated into gallstones.

The role of bacteria extends beyond enzymatic activity. Several studies have shown that many biliary bacteria produce mucus, a feature found to be even more strongly associated with pigmented stone formation than enzymatic activity alone. This mucus facilitates biofilm formation, creating complex structures that protect microorganisms from antibiotics and contribute to persistent, difficult-to-eradicate biliary infections [50]. In patients with choledocholithiasis, a high prevalence of mucus- and biofilm- producing bacteria has been observed. These microorganisms may therefore play a role not only in stone formation but also in biliary obstruction and the development of systemic infections such as bacteremia [51,52]. Current evidence indicates that the phyla most frequently detected in the biliary tract of individuals with choledocholithiasis include *Firmicutes*, *Proteobacteria*, *Bacteroidota* and *Actinobacteriota*, although their relative abundance may vary across studies due to methodological and clinical heterogeneity [53,54].

Among the microorganisms identified in bile samples from patients with cholelithiasis, *E. coli* emerges as the predominant isolate, followed by *Klebsiella* spp. and *Enterobacter* spp. [55]. Aerobic bacteria represent the most commonly encountered pathogens in biliary infections, with frequent isolates including *E. coli*, *Klebsiella* spp., *E. faecalis*, *Streptococcus* spp., *Pseudomonas* spp., and *Salmonella* spp. [56].

Although less frequently reported, anaerobic organisms such as *Clostridium* spp., *B. fragilis*, and *Propionibacterium* spp. have also been detected in certain clinical contexts [42,57]. The prevalence of biliary tract infections has been shown to increase with advancing age, particularly in individuals over 60 years and in those with acute cholecystitis. Interestingly, neither gender nor the number of choledochal stones appears to significantly influence the probability of bacterial colonization in bile cultures [52]. A recent study has drawn attention to specific species as *P. merdae*, *S. dysgalactiae* and *S. agalactiae* which harbors the *uidA* and *pldA* genes encoding the aforementioned enzymes [45]. This microorganism appears to play a critical role in the formation of pigmented gallstones (PGS). Additionally, it participates in bile acid metabolism, generating isolithocholic acid, a derivative with potent antibacterial activity against drug-resistant Gram-positive pathogens [45]. This promotes the selective enrichment of Gram-negative bacteria in bile [58]. The reported Gram-negative bacteria, through the secretion of extracellular polymeric substances (EPS), contribute to biofilm development, which becomes an integral part of the gallstone structure [59].

Gallstones are classified into three main categories: cholesterol stones, black pigment stones, and brown pigment stones. Bacteria have been detected in 75% of pigment stones, 76% of mixed stones, and 20% of cholesterol stones. According to Kose et al., the composition of the gut microbiota may influence the type of gallstones formed (cholesterol, pigment, or mixed) [59]. Bacterial characteristics have been shown to influence the process of gallstone formation. In particular, various bacterial species play a pivotal role in the formation of brown pigment stones by actively participating in the biochemical pathways leading to lithogenesis [59].

Among these, *Actinomyces* is one of the most studied taxa, known for its ability to form biofilms and produce the enzyme β-glucuronidase. *Achromobacter* spp. have been associated with pigment production, including a red pigment derived from the oxidation of L-tryptophan, potentially responsible for the brown coloration observed in some stones [60]. *E. coli* is another major producer of β-glucuronidase and plays a well-established role in the pathogenesis of pigment lithiasis.

Additional bacteria frequently isolated from infected bile samples include *K. pneumoniae*, *Salmonella* spp., and *P. aeruginosa*. These organisms contribute to lithogenesis through their metabolic activity [59]. Anaerobic bacteria, such as *C. perfringens*, *B. fragilis*, and *Eubacterium* spp., are primarily involved in the degradation of biliary phospholipids, a process that releases lipid components conducive to the nucleation of crystals.

Cholesterol stones, traditionally considered sterile, have recently been associated with specific bacterial signatures. Recent studies demonstrated that approximately one-third of bacterial strains isolated from cholesterol gallstones are capable of producing both β-glucuronidase and phospholipase, suggesting that microbial activity may influence cholesterol stone formation under certain conditions [61,62]. A study from 2013 reported *L. raffinolactis*, *P. acnes*, *A. flavithermus* and *C. segnis* as bacterial species potentially associated with the presence of cholesterol gallstones [63].

Recent studies have identified Gram-positive bacteria, such as *Bacillus* and *Alcaligenes*, within cholesterol stones. These organisms are also implicated in biofilm formation and the production of lipolytic enzymes such as phospholipase A2. This enzyme may contribute to the formation of calcium palmitate, which serves as a nidus for cholesterol crystallization [64].

Furthermore, an enrichment of bacteria belonging to the order *Desulfovibrionales* has been observed in patients with cholesterol gallstones with *D. fairfieldensis*, *piger*, *desulfuricans*, and *vulgaris* identified as the major species to be found. These bacteria can metabolize sulfur-containing compounds such as taurine, producing hydrogen sulfide (H_2_S). H_2_S significantly affects bile acid metabolism by reducing bile acid synthesis. Additionally, H_2_S promotes the growth of bacteria capable of 7α-dehydroxylation of primary bile acids, leading to the formation of secondary bile acids such as deoxycholic acid (DCA) [62]. Increased DCA levels render bile more hydrophobic, enhancing intestinal cholesterol absorption and contributing to hepatic cholesterol overload. This, in turn, increases biliary cholesterol secretion, resulting in bile supersaturation and promoting cholesterol stone formation. Moreover, such microbial-mediated inhibition may reduce cholesterol excretion into bile, further supporting cholesterol crystallization and stone formation [65].

Overall, pigment stones are primarily associated with Gram-negative bacteria involved in bilirubin deconjugation and biofilm formation. In contrast, cholesterol stones exhibit a more diverse microbial composition, often including Gram-positive bacteria that contribute to biofilm formation and the production of lipolytic enzymes. These differences support distinct pathogenetic mechanisms in the formation of the two main types of gallstones. To provide a concise and systematic overview of disease-associated biliary microbiota, the main microorganisms identified across different pathological conditions and their putative roles are summarized in Table 2.

### 3.3. Risk of Gallstone Recurrence in Relation to Microbiota, Bacterial Enzymes, and Biofilm Formation

Numerous studies have shown that gallstones, especially pigment stones, harbor bacteria embedded in biofilms‘ three-dimensional structures composed of microbial aggregates enclosed within an extracellular matrix made of polysaccharides, proteins, and DNA. This matrix provides protection against antibiotics and host immune responses, contributing to the persistence of infection [66,67].

Biofilm formation plays a pivotal role in both the pathogenesis and recurrence of gallstones, by creating a microenvironment that favors the nucleation and growth of biliary crystals [62]. A key element in biofilm development is the production of bacterial mucus (or “slime”), a viscous substance that facilitates bacterial adhesion to epithelial surfaces and to the stones themselves [51]. From a clinical perspective, the interaction between biofilm structure, mucus production, and the activity of bacterial enzymes such as β-glucuronidase and phospholipase has a major impact on the severity of infection and the risk of recurrence. Bacteria with low slime production (LowSL) are more frequently isolated from both bile and blood cultures compared to high slime-producing strains (HighSL), suggesting that less viscous biofilms may facilitate systemic bacterial dissemination [51].

According to a study by Stewart et al. no high-slime-producing bacteria were recovered from blood cultures. Moreover, the incidence of bactibilia was significantly higher in patients with LowSL bacteria (79%) compared to those with HighSL bacteria (42%, *p* = 0.028), and bacteremia was observed in 22% of LowSL cases versus 0% in HighSL cases [51]. Similarly, the presence of β-glucuronidase/phospholipase-producing bacteria (bGPhL+) was associated with an increased incidence of bactibilia (86% vs. 61%, *p* = 0.004) and showed a trend toward higher bacteremia (19% vs. 5%, *p* = 0.091), compared to patients without these enzymes [51].

The combined effect of low slime production and the presence of β-glucuronidase/phospholipase enzymes proved to be particularly virulent. Patients harboring LowSL and β-glucuronidase/phospholipase-producing bacteria had the highest incidence of bactibilia (90%) and bacteremia (29%), compared to all other groups [63]. In contrast, patients with HighSL bacteria lacking enzymatic activity exhibited the lowest infection rates (bactibilia 20%, bacteremia 0%). These findings suggest that low-viscosity biofilms facilitate bacterial detachment and systemic spread, while the enzymatic activity of β-glucuronidase and phospholipase alters bile composition, promoting the formation of pigment stones [52,68,69].

The risk of gallstone recurrence seems to be strongly influenced by the presence of low-slime-producing bacteria, the expression of lithogenic enzymes, and the capacity to form structured biofilms. Thus, the management of gallstone disease should go beyond surgical and antibiotic therapies to include strategies aimed at modulating the biliary microbiota, inhibiting lithogenic enzyme activity, and disrupting biofilm architecture in order to prevent disease recurrence.

## 4. Pathogens Isolated from Bile: Antibiotic Resistance Patterns

### 4.1. Most Frequent Isolates

During infectious processes involving the biliary tract, specific pathogens emerge as dominant agents, distinct from the commensal microbiota identified under non-pathological conditions. The most commonly isolated microorganisms from bile cultures in patients with biliary infections are *E. coli*, *K. pneumoniae*, *Enterococcus* spp., and *P. aeruginosa* [70,71,72]. *E. coli* consistently ranks as the most prevalent Gram-negative isolate, particularly in community-acquired cholangitis and cholecystitis [70]. *K. pneumoniae* follows closely and is often associated with severe cases, especially in patients with hepatobiliary malignancies or a history of biliary interventions [72]. Among Gram-positive organisms, *E. faecalis* and *E. faecium* are frequently recovered. Their presence is more prominent in patients with previous antibiotic exposure, biliary stenting, or nosocomial infections [71,72]. *P. aeruginosa* and other non-fermentative Gram-negative bacilli are predominantly isolated from patients with complicated or hospital-acquired biliary infections, often reflecting prior broad-spectrum antibiotic use and biliary drainage procedures [70]. The spectrum of isolates varies according to geographic region, underlying comorbidities, and patterns of local antimicrobial resistance. Mixed infections with anaerobes, particularly *Bacteroides* spp., may also occur but are less frequently documented due to limitations in standard culture techniques.

### 4.2. Mechanisms of Resistance in Biliary Pathogens

The increasing incidence of antimicrobial resistance among biliary pathogens poses a major therapeutic challenge, particularly in the setting of multidrug-resistant (MDR) strains.

- Extended-Spectrum Beta-Lactamase (ESBL) Production: Many *E. coli* and *K. pneumoniae* strains isolated from bile produce ESBLs, conferring resistance to third-generation cephalosporins and monobactams [71,72]. ESBL production is particularly concerning as it limits empirical treatment options and is associated with worse clinical outcomes. ESBL-producing isolates often harbor co-resistances to fluoroquinolones and aminoglycosides.
- Carbapenem Resistance and Carbapenemases: Carbapenem-resistant *K. pneumoniae* (CRKP) is an emerging pathogen in biliary infections, especially in nosocomial settings [71]. Carbapenem resistance is mediated by enzymes such as KPC (Klebsiella pneumoniae carbapenemase) and NDM (New Delhi metallo-β-lactamase), which hydrolyze carbapenems and other β-lactams. These organisms are often resistant to nearly all available antibiotics except polymyxins and newer β-lactam/β-lactamase inhibitor combinations.
- Vancomycin-Resistant Enterococci (VRE): *E. faecium* isolates in biliary infections increasingly display resistance to vancomycin, primarily through the acquisition of *vanA* and *vanB* gene clusters [72]. VRE are of particular concern due to limited treatment options, often requiring the use of linezolid or daptomycin.
- Pseudomonas aeruginosa Multidrug Resistance: *P. aeruginosa* displays intrinsic resistance mechanisms, including the overexpression of efflux pumps (e.g., MexAB-OprM), porin loss (OprD), and β-lactamase production. MDR *P. aeruginosa* strains can be resistant to carbapenems, aminoglycosides, and fluoroquinolones, complicating the selection of appropriate empiric therapy [71].
- Clinical Impact of Resistance: Infections with resistant biliary pathogens are associated with higher rates of treatment failure, longer hospital stays, increased rates of recurrent cholangitis, and higher mortality. Empirical antibiotic therapy must therefore be guided by local resistance patterns and adjusted based on microbiological findings whenever possible [71].

The growing burden of ESBL-producing *Enterobacterales*, carbapenemase-producing *K. pneumoniae*, VRE, and MDR *P. aeruginosa* highlights the need for antimicrobial stewardship strategies, targeted empirical therapy, and early drainage procedures to improve clinical outcomes.

### 4.3. Relevance for Empirical Antibiotic Therapy and Perioperative Management

ERCP is a therapeutic yet invasive endoscopic procedure that carries a measurable risk of infectious complications (post-ERCP cholangitis, bacteremia, and, less commonly, liver abscess). These infections often lead to higher morbidity, prolonged hospital stay and increased healthcare costs, hinting a crucial role for pre-procedural planning [73]. Current guidelines from ESGE, ASGE and the Tokyo Guidelines support a selective, risk-adapted use of antibiotics rather than routine prophylaxis for ERCP procedures. Antibiotics are generally not recommended when complete biliary toilette is obtained. In contrast, prophylaxis becomes appropriate when there is a high likelihood of incomplete drainage, such as in hilar obstructions, primary sclerosing cholangitis or complex strictures, or in severely immunocompromised patients [42,74,75].

Prophylactic coverage is also advised when cholangioscopy is planned, given the increased manipulation of the biliary system. When clinically indicated antibiotics covering enteric Gram-negative and enterococcal organisms are recommended, and therapy should be continued when drainage remains incomplete. In this context, whenever feasible, bile cultures should be obtained during ERCP to guide de-escalation. The duration of antibiotic therapy should be tailored according to the severity and culture results to preserve antimicrobial stewardship [75,76]. Hence, a correct perioperative management requires a balance between minimizing infectious risk and avoiding overtreatment, considering both patient risk factors and epidemiological data.

## 5. Interplay Between Biliary and Intestinal Microbiota

### 5.1. Evidence for Microbiota Cross-Talk Along the Gut–Liver–Biliary Axis

**Figure 3:**
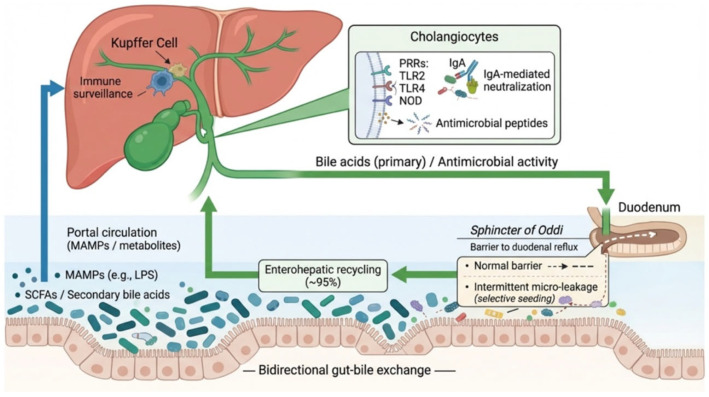
The gut–liver–biliary axis: a bidirectional interface between microbiota, bile acids, and host immunity. The biliary tract participates in a dynamic bidirectional exchange with the intestinal microbiota along the gut-liver-biliary axis. Primary bile acids synthesized by hepatocytes exert antimicrobial activity and shape intestinal microbial communities, while gut microbiota transforms bile acids into secondary metabolites that return to the liver through enterohepatic circulation. Microbial products and metabolites reaching the liver via the portal vein modulate hepatic and biliary immune responses. Cholangiocytes contribute to mucosal defense through pattern recognition receptors (e.g., TLR2, TLR4, NOD-like receptors), antimicrobial peptide secretion, and IgA-mediated immune exclusion. The sphincter of Oddi acts as a physiological barrier limiting duodenal reflux, although intermittent micro-leakage may permit controlled microbial exposure under physiological conditions. Created in BioRender. Restaneo, L. (https://www.figurelabs.ai, accessed on 3 March 2026) is licensed under CC BY 4.0.

Compelling evidence supports the existence of a tightly interconnected and bidirectional microbiota cross-talk along the gut–liver–biliary axis, in which intestinal microbial communities, bile acid metabolism, immune signaling, and hepatobiliary physiology are functionally integrated [26,77,78,79]. Anatomically, the liver is continuously exposed to gut-derived microbial products via the portal circulation, including lipopolysaccharide (LPS), bacterial DNA, and other pathogen-associated molecular patterns (PAMPs), which shape hepatic innate immune responses and influence biliary homeostasis [80,81,82]. Conversely, the liver exerts a regulatory effect on gut microbiota composition through the secretion of bile acids, antimicrobial peptides, and secretory IgA into the biliary tract and intestine, thereby establishing a dynamic feedback loop between the gut and hepatobiliary system [83,84,85]. Bile acids play a central signaling role within this axis, as primary bile acids synthesized in hepatocytes are extensively modified by intestinal microbiota into secondary bile acids. These, in turn, modulate microbial ecology, intestinal barrier integrity, and host metabolic and inflammatory pathways through activation of nuclear and membrane receptors such as farnesoid X receptor (FXR) and Takeda G protein–coupled receptor 5 (TGR5) [23,86,87,88,89]. Disruption of gut microbial homeostasis has been shown to alter bile acid composition and signaling, promote intestinal permeability, and facilitate microbial translocation and retrograde colonization of the biliary tree. These observations are supported by culture-independent sequencing studies demonstrating significant taxonomic overlap between gut and biliary microbiota, including genera such as *Escherichia*, *Enterococcus*, *Klebsiella*, *Bacteroides*, and *Clostridium* [18,90,91]. At the immune level, hepatocytes, Kupffer cells, and cholangiocytes express pattern recognition receptors enabling the sensing of microbial ligands, thus triggering inflammatory cascades that contribute to cholangiocyte dysfunction, biliary inflammation, gallstone formation, and cholestatic liver disease [92,93,94,95]. Experimental and translational studies further corroborate this cross-talk. The modulation of gut microbiota through antibiotics, probiotics, bile acid receptor agonists may result in significant changes in bile composition, biliary microbial profiles, and susceptibility to hepatobiliary diseases, highlighting the clinical relevance of the gut–liver–biliary axis in both healthy and ill subjects. Figure 3 summarized the interplay of the gut–liver–biliary axis.

### 5.2. The Relation Between Dysbiosis and Immune and Antimicrobial Resistance Responses

Over the past decades, a crucial interplay between gut microbiota and both innate and adaptative host immunity response has been reported, suggesting a potential role of this mutual relationship in antimicrobial resistance [96]. Interactions between gut microbiota and innate immune system are governed by feedback loops that primarily occur at the superficial layers of the intestinal mucosa where the recognition of microorganisms by pattern recognition receptors (PRRs) triggers transcriptional responses and the secretion of effector molecules. Conversely, the adaptive immune response involves the secretion of IgA and the regulation of T cells [96]. When alterations in the microbial ecosystem result in loss of beneficial species, overgrowth of pathobionts and reduction in the alpha diversity. In this condition, known as dysbiosis, both innate and adaptive immune responses appear to be affected [97]. Dysbiosis is implicated in the disruption of the production of SCFAs by commensal bacteria, leading to impaired gut mucosal integrity, as well as in the modulation of inflammasome-signaling mediated by Toll-like receptors and the NOD-like receptor NLRP6, whose mechanisms perpetrate the inflammatory state [97,98,99,100]. Collectively, these mechanisms demonstrate that the gut microbiota plays a central role in regulating the host immune response and that its alteration can predispose the host to inflammatory disorders and subsequently to altered antimicrobial resistance patterns. Antimicrobial resistance is a major global health concern, and the gut microbiota has emerged as a critical reservoir and amplifier of resistance mechanisms. The intestinal environment, due to its high bacterial density and biofilm-forming capacity, provides an ideal ecosystem for the selection and exchange of antibiotic resistance genes, also known as *gut resistome* [101]. Commensal and opportunistic bacteria accumulate resistance determinants and can transfer them to pathogens through horizontal gene transfer (HGT) mechanisms such as conjugation, transformation, and transduction [102]. Several specific processes contribute to this phenomenon. For instance, *Bacteroides* spp. have been shown to release β-lactamases via outer membrane vesicles, conferring protection to microbes against β-lactam antibiotics [103]. Moreover, quorum sensing (QS), a bacterial communication system regulating gene expression, plays a pivotal role in coordinating biofilm formation and modulating resistance [104]. Through QS signaling, bacteria can upregulate efflux pumps, thereby enhancing multidrug resistance [105]. Environmental and drug exposure can modulate the *gut resistome* and select for resistant taxa [106]. In this context dysbiosis further exacerbates this issue, reducing microbial diversity and weakening colonization resistance, therefore facilitating the overgrowth of resistant pathogens [107,108].

### 5.3. Potential of Fecal or Bile Microbiota Profiling as Diagnostic/Prognostic Markers

The gut–biliary axis has emerged as a potential contributor to the pathogenesis of different biliary diseases [109,110]. However, research exploring whether specific microbial signatures in bile or feces could serve as diagnostic or prognostic biomarkers in infectious and lithiasic biliary disorders remains at an early stage.

In patients with biliary stone disease, elevated levels of secondary bile acids have been observed [67,111]. Gut microbiota contributes to the transformation of primary into secondary bile acids through 7-dehydroxylation, a function performed by specific bacterial taxa such as *Clostridia* [112]. These bacteria enhance hydrogen sulfide (H_2_S) production and stimulate 7α-dehydroxylating bacterial populations, thereby promoting secondary bile acid synthesis. This cascade ultimately leads to increased intestinal cholesterol reabsorption and its subsequent excretion into bile, promoting gallstones formation [67]. The elevated fecal concentrations of bile acids observed in patients with gallstones seems to elicit a compositional shift in the gut microbiota, which may represent a potential diagnostic biomarker for this condition. Notably *Oscillospira* seems to benefit from the elevated concentrations of biliary acids in the colon, whereas the genus *Roseburia* exhibits an inverse correlation with bile acid concentrations [113]. Literature on biomarkers for acute cholangitis and cholecystitis is currently limited. A prospective study conducted in Japan suggested a potential correlation between higher levels of corisin, a microbiome-derived pro-apoptotic peptide found in bile and plasma samples, and clinical progression of acute cholangitis, hinting a possible role as a biomarker [114]. The available data are only preliminary, highlighting the need for larger, standardized studies. Further research is therefore needed to assess the clinical relevance and potential of fecal and bile microbiota profiling in infectious diseases.

## 6. Clinical Implications and Future Directions

The recognition of a structured and functionally relevant biliary microbiota has relevant implications for the clinical management of biliary infections and their complications. However, evidence regarding the resident bile microbiota in healthy subjects is still accumulating, and further research is still needed to reach a deeper understanding of its role. Indeed, available studies are burdened by methodological limitations, including small sample sizes, various and non-standardized bile collection techniques. A microbiota-informed approach to antibiotic stewardship may improve empirical antibiotic selection and support timely de-escalation once microbiological data become available, in line with current international recommendations for acute biliary infections [42]. Conventional culture-based diagnostics remain essential but may underestimate microbial diversity and fail to capture fastidious or biofilm-associated organisms, as highlighted by culture-independent studies of the biliary microbiome [18]. Moreover, culture-based diagnostics carry an intrinsic high risk of contamination, which may, in worst-case scenarios, even mislead therapeutic choices. In selected high-risk clinical scenarios—such as recurrent cholangitis, previous biliary instrumentation, or suspected colonization by multidrug-resistant organisms—the integration of molecular microbiological techniques with standard cultures may refine etiological attribution and improve therapeutic precision [18,42].

Beyond antimicrobial therapy, the modulation of the biliary–intestinal microbial axis represents a potential therapeutic avenue. Experimental and translational studies support a mechanistic link between gut microbiota composition, bile acid metabolism, and gallstone formation, providing a biological rationale for interventions aimed at microbiota modulation, including dietary strategies, probiotics, or bile-acid-targeted therapies [66]. Although direct interventional evidence in human biliary disease remains limited, biofilm biology appears particularly relevant from a clinical perspective. Gallstones frequently harbor bacteria embedded within biofilms, and biofilm-related phenotypes have been associated with increased infection severity and bacteremia, suggesting that strategies targeting biofilm formation or persistence may help reduce infectious complications and disease recurrence [51]. Major research gaps remain, including the need for longitudinal studies, standardized sampling protocols for low-biomass bile specimens, and mechanistic investigations capable of distinguishing causality from association [42,66]. Moreover, high quality research to investigate the efficacy of the currently available therapeutic armamentarium to influence the bile microbiota in both healthy and non-infectious disease is needed. A deeper knowledge of the gut–liver–biliary axis may also lead to the development of new therapies or repurposing of already existent ones, hitting targets different from pathogens such as microfilm and mucus, bile acids and bile compositions.

Ultimately, the integration of biliary microbiota profiling with clinical, procedural, and microbiological data may support personalized risk stratification, surgical planning, and peri-procedural management, consistent with a risk-adapted approach to antibiotic prophylaxis and biliary drainage endorsed by current endoscopy guidelines [73,75].

## 7. Conclusions

Accumulating evidence challenges the traditional concept of biliary sterility and supports the presence of a resident biliary microbiota with potential functional relevance in health and disease. Alterations in biliary microbial composition have been associated with gallstone formation, biliary tract infections, and antimicrobial resistance patterns that complicate clinical management. The bidirectional cross-talk along the gut–liver–biliary axis, mediated by enterohepatic circulation, bile acid signaling, immune regulation, and biofilm formation, provides a coherent framework to interpret how microbial dysbiosis may contribute to lithogenesis, inflammation, and susceptibility to infection.

Within this evolving paradigm, the biliary microbiota emerges as a potential component of precision medicine in hepatobiliary disorders, complementing clinical, laboratory, imaging, and microbiological data to refine diagnostic, prognostic, and therapeutic strategies. Translating these insights into routine clinical practice will require close interdisciplinary collaboration among gastroenterologists, hepatobiliary surgeons, microbiologists, and infectious disease specialists, together with methodological standardization and prospective studies designed to support implementation in real-world clinical settings.

## Figures and Tables

**Table 1:** Representative microbial taxa identified in the healthy biliary tract.

Taxonomic Level	Dominant Groups	Notable Features
Phyla	*Firmicutes*, *Proteobacteria*, *Bacteroidetes*, *Actinobacteria*	Bile tolerance, adaptation to low-oxygen, high-surfactant environments
Genera	*Streptococcus*, *Escherichia-Shigella*, *Prevotella*, *Veillonella*, *Enterococcus*	Facultative anaerobes; resistance to bile acids; potential biofilm formation
Other Components	Fungi (*Candida* spp.), bacteriophages (preliminary findings)	Emerging evidence; potential roles in microbiota stability

**Table 2:** Representative microorganisms associated with biliary diseases.

Pathological Condition	Microorganisms (Genus/Species)	Type	Pathophysiological Role/Notes
Acute cholecystitis/cholangitis	*Escherichia coli*; *Klebsiella pneumoniae*; *Pseudomonas aeruginosa*; *Enterococcus faecalis*/*faecium*; *Candida* spp.	Gram-negative, Gram-positive, fungi	Ascending infection; bactibilia; potential sepsis; antimicrobial resistance patterns relevant
Cholelithiasis (general)	*E. coli*; *Klebsiella* spp.; *Enterobacter* spp.; *Streptococcus* spp.; *Salmonella* spp.	Mainly aerobic bacteria	Colonization of bile; involvement in infection and lithogenesis
Pigment gallstones	*E. coli*; *Actinomyces*; *Achromobacter* spp.; *Clostridium* spp.; *Bacteroides fragilis*	Gram-negative and anaerobes	β-glucuronidase production; bilirubin deconjugation; biofilm formation
Cholesterol gallstones	*Lactococcus raffinolactis*; *Propionibacterium acnes*; *Anoxybacillus flavithermus*; *Clostridium segnis*; *Bacillus*; *Alcaligenes*	Mainly Gram-positive	Phospholipase activity; biofilm; contribution to cholesterol crystallization
Choledocholithiasis	*Firmicutes*; *Proteobacteria*; *Bacteroidota*; *Actinobacteriota* (phyla)	Mixed microbiota	Dysbiosis with variable abundance; associated with obstruction and inflammation
Biofilm-associated infection/recurrence	EPS-producing bacteria (various Gram-negatives); β-glucuronidase/phospholipase producers	Mixed	Biofilm formation; persistence; increased recurrence and bacteremia risk
Sulfur-metabolizing bacteria (cholesterol stones)	*Desulfovibrio* spp. (*D. fairfieldensis*, piger, desulfuricans, vulgaris)	Gram-negative anaerobes	H_2_S production; altered bile acid metabolism; increased cholesterol saturation

## Data Availability

No new data were created or analyzed in this study. Data sharing is not applicable.
